# Synthetic CpG oligonucleotides induce a genetic profile ameliorating murine myocardial I/R injury

**DOI:** 10.1111/jcmm.13616

**Published:** 2018-04-19

**Authors:** Tobias Hilbert, Paul Markowski, Stilla Frede, Olaf Boehm, Pascal Knuefermann, Georg Baumgarten, Andreas Hoeft, Sven Klaschik

**Affiliations:** ^1^ Department of Anesthesiology and Intensive Care Medicine University Hospital Bonn Bonn Germany; ^2^ Department of Anesthesiology and Intensive Care Medicine Gemeinschaftskrankenhaus Bonn St. Elisabeth ‐ St. Petrus ‐ St. Johannes gGmbH Bonn Germany; ^3^ Department of Anesthesiology and Intensive Care Medicine Johanniter Hospital Bonn Bonn Germany

**Keywords:** 1668, CpG oligonucleotide, IL‐10, ischaemia/reperfusion injury, microarray analysis, myocardial infarction, pre‐conditioning

## Abstract

We previously demonstrated that pre‐conditioning with CpG oligonucleotide (ODN) 1668 induces quick up‐regulation of gene expression 3 hours post‐murine myocardial ischaemia/reperfusion (I/R) injury, terminating inflammatory processes that sustain I/R injury. Now, performing comprehensive microarray and biocomputational analyses, we sought to further enlighten the “black box” beyond these first 3 hours. C57BL/6 mice were pretreated with either CpG 1668 or with control ODN 1612, respectively. Sixteen hours later, myocardial ischaemia was induced for 1 hour in a closed‐chest model, followed by reperfusion for 24 hours. RNA was extracted from hearts, and labelled cDNA was hybridized to gene microarrays. Data analysis was performed with BRB ArrayTools and Ingenuity Pathway Analysis. Functional groups mediating restoration of cellular integrity were among the top up‐regulated categories. Genes known to influence cardiomyocyte survival were strongly induced 24 hours post‐I/R. In contrast, proinflammatory pathways were down‐regulated. Interleukin‐10, an upstream regulator, suppressed specifically selected proinflammatory target genes at 24 hours compared to 3 hours post‐I/R. The IL1 complex is supposed to be one regulator of a network increasing cardiovascular angiogenesis. The up‐regulation of numerous protective pathways and the suppression of proinflammatory activity are supposed to be the genetic correlate of the cardioprotective effects of CpG 1668 pre‐conditioning.

## 1 INTRODUCTION

Myocardial ischaemia can be induced either artificially during aortic cross‐clamping in cardiac surgery, or naturally during acute occlusion of coronary arteries due to plaque rupture and subsequent thrombosis (myocardial infarction, MI). Tissue perfusion is re‐established by either releasing the aortic clamp or revascularization of the occluded coronary vessel during, for example, percutaneous coronary intervention. Reperfusion of the tissue itself is known to cause additional tissue damage.^1^ As the extent of ischaemia and reperfusion (I/R) injury negatively influences functional recovery and subsequently patient morbidity and mortality, several strategies to reduce I/R injury have been proposed over the years. For a detailed overview, we refer to some excellent recent review publications.^2^, ^3^, ^4^, ^5^ One concept for reducing I/R damage is the application of physical (repetitive hypoxic stimulation, hypo‐/hyperthermia), pharmacological (eg anaesthetics or opioids) or immunological effectors, such as ligands for Toll‐like receptors (TLRs). Both pre‐ and post‐conditioning with TLR ligands can trigger a myocardial inflammatory response, which may potentially diminish the damage induced by I/R.^5^, ^6^ Successful implementation of this approach in a murine I/R model has been demonstrated by our group using ligands for TLR 2, 4 and 9.^7^, ^8^, ^9^ In particular, synthetic TLR9 ligands, such as CpG oligodeoxynucleotides (CpG ODNs), prompt a strong innate immune response with only moderate side effects in the human organism.^10^ Hence, they are suitable for use as immunostimulatory agents as well as vaccine adjuvants in clinical trials. Their potential to stimulate the innate immune system depends on the cytosine and guanine (CG) triphosphate deoxynucleotide motif as well as on the degree of methylation.^11^ In a previous study, we could demonstrate that the intraperitoneal (ip) injection of C57BL/6 wild‐type mice with the CpG ODN 1668 thioate significantly boosted levels of inflammatory serum mediators and concomitantly raised the number of immune cells in blood and the spleen.^12^ In addition, this intervention up‐regulated gene expression of myocardial pattern recognition receptors. After myocardial ischaemia and reperfusion induced in a closed‐chest model 16 hours after pre‐conditioning, infarct size as well as rates of failure of cardiac function was reduced by up to 75%, which did not occur in the control groups. Protein and RT‐PCR analyses (performed 3 hours after start of reperfusion) strongly suggested that an excessive CpG‐dependent up‐regulation of interleukin‐10 (IL‐10) was one key factor in reducing infarct size and improving cardiac function. This was further supported by the fact that blocking IL‐10 prevented the cardioprotection afforded by CpG pre‐conditioning. Interestingly, genome profiling revealed alterations in the level of activity of IL‐10‐dependent genes as well, considered to elicit suppressive networks that terminate the inflammatory processes that sustain I/R injury. Furthermore, CpG pre‐conditioning together with I/R induced massive escalation of gene expression in general, highly exceeding what was observed after CpG priming or I/R alone. Bioinformatic analysis revealed that genes mediating the inflammatory response or immune cell trafficking were among the most prominently up‐regulated functional groups.

Despite these findings, such striking changes in gene regulation have been described incompletely, as pathway and network analyses have only been performed over a short period of time, that is up to 3 hours, following I/R. It has been demonstrated that, on the one hand, both harmful and protective mechanisms are distinctly altered at 24 hours after I/R, suggesting that this vulnerable time‐point may be a critical period for any interventional therapeutic strategy.^13^, ^14^, ^15^, ^16^ On the other hand, the protective time span of immunologic pre‐conditioning strategies to minimize I/R injury has been shown to be most pronounced in the 24‐72 hours following administration of TLR ligands.^17^, ^18^ This prompted us to extend the observational period and perform comprehensive microarray and biocomputational analyses at a later time‐point to illuminate the protective mechanisms of CpG pre‐conditioning during this temporal “black box.” We wondered whether long‐term effects, such as the observed restoration of cardiac function mediated by CpG, might have a preceding correlate at the level of gene expression. Our results may help further elucidate the protective molecular mechanisms of CpG ODN‐mediated amelioration of myocardial I/R injury.

## 2 MATERIALS AND METHODS

All mice were handled in accordance with the Guide for Use and Care of Laboratory Animals (NIH publication No. 85‐23, revised 1996). Protocols were approved by the local animal authority (protocol no. AZ 8.87‐51.04.20.09.392; LANUV, Recklinghausen, Germany).

### 2.1 Surgical techniques, experimental groups and protocols

**Figure 1:**
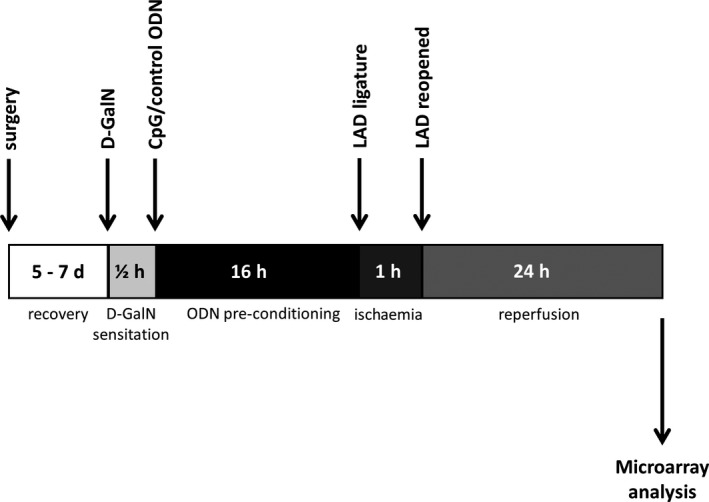
Time course of experimental interventions and assessments. Nonlinear scale for better identification of various interventions. ODN = CpG/control oligodeoxynucleotide, LAD = left anterior descending artery

Ten‐ to 12‐week‐old male C57BL/6 mice (Charles River Laboratories, Sulzfeld, Germany) were included. To prevent an inflammatory reaction due to surgical trauma, a chronic closed‐chest model of myocardial I/R with left parasternal incision was applied. Surgical procedures have been described and depicted in detail in a previous publication.^19^ In brief, in mice anesthetized with isoflurane (2.5 Vol%; Forene, Abbott GmbH, Wiesbaden, Germany) and mechanically ventilated (respiratory rate 105*min^−1^, 200 μL tidal volume; Minivent, Hugo Sachs Elektronik‐Harvard Apparatus GmbH, March‐Hugstetten, Germany), a left parasternal incision was performed and an 8‐0‐prolene suture was passed underneath the left anterior descending artery (LAD). Both ends of the suture were threaded through a polyethylene tube and were tightened to confirm correct position by emerging paleness of the distal myocardium. Afterwards, both ends of the suture were released again, formed into a knot, placed subcutaneously, and the chest was closed with 6‐0 sutures. Analgesia was obtained by application of buprenorphine (0.1 mg/kg s.c.), and mice were allowed to recover from anaesthesia. After 5‐7 days, all mice were treated with an ip dose of d‐Galactosamine N (DGalN, 0.2 mg/g body weight (BW), Roth, Karlsruhe, Germany) to slow down the hepatic degradation of CpG ODNs (Figure 1).^20^ In control experiments, DGalN alone did not induce an inflammatory response (data not shown). Animals were randomized to ip received either a stimulatory CpG ODN (1668 thioate; 5′‐TCCATGACGTTCCTGATGCT; TIB Molbiol, Berlin, Germany; 0.25 nmol/g BW), a non‐CG containing control ODN (1612 thioate; 5′‐GCTAGATGTTAGCGT; TIB Molbiol; 0.25 nmol/g BW) or PBS as control. In previous experiments, the dosage of CpG ODN 1668 had been tested. Pre‐conditioning was performed 16 hours before I/R, and this was chosen because our previous experiments showed that pre‐conditioning with the TLR4 ligand lipopolysaccharide results in a reduction in infarct size after this time span, and that priming with CpG ODN applied under the same conditions (interval and concentration) as used here attenuated cardiac hypertrophy induced by transverse aortic constriction.^8^, ^21^ Myocardial infarction was then induced for 1 hour (for time course see Figure 1). Mice were sedated with propofol (1% in 0.2 mL; 2 mg ip). Electrocardiogram (ECG; PowerLab, ADInstruments GmbH, Spechbach, Germany) was recorded. The original skin incision was reopened, and the subcutaneously placed thread was manipulated without opening the chest. Thereafter, tension was carefully applied on the loop to achieve an ischaemia, operationalized here as a significant ST‐elevation. One hour after ischaemia, the loop was released and reperfusion induced the recession of the ST‐segment elevation. Finally, the skin was closed, followed by 24 hours of reperfusion.

### 2.2 RNA extraction and production of labelled cDNA

Total RNA was isolated according to a standard protocol (Qiagen, Hilden, Germany). RNA (20 μg) was reverse‐transcribed using 3 μL 10× of first‐strand buffer (Stratagene, La Jolla, CA, USA), 2 μL (5 μg) of anchored oligo (dT) (Thermo Fisher Scientific, Waltham, MA, USA), 150U of RT enzyme (StrataScript HC RT; Stratagene), 2 μL 20× of aminoallyl‐dUTP/dNTP mix and 3 μL of 0.1 mol/L DTT in a final volume of 30 μL at 42°C for 90 minutes. A reference mouse RNA sample (Stratagene) was processed in parallel. Both cDNAs were purified using the MinElute PCR Purification Kit (Qiagen). Complementary DNA (cDNA) (10 μL) was labelled with Cy5‐(sample cDNA) or Cy3‐(universal reference cDNA) reactive dyes (Amersham Biosciences, Piscataway, NJ, USA), diluted in 5 μL DMSO plus 1.7 μL of 1 mol/L NaHCO_3_ for 90 minutes in the dark. Labelled cDNA was purified using the MinElute PCR Purification Kit (Qiagen).

### 2.3 Microarray hybridization

Cy3‐labelled reference and Cy5‐labelled sample cDNAs (10 μL each) were combined, denatured by heating for 2 minutes at 98°C and mixed with 36 μL of hybridization solution at 42°C (Ambion, Austin, TX, USA). Murine microarrays (NimbleGen, Roche, Basel, Switzerland) were overlaid with this solution and hybridized for 18 hours at 42°C using an actively mixing MAUI hybridization system (BioMicro Systems, Salt Lake City, UT, USA). Post‐hybridization, the arrays were washed in 1× SSC/0.05% SDS and 0.1× SSC, centrifuged to remove the remaining liquid with unbound cDNA and dried. Intensity values were generated using an array scanner (NimbleGen). Data were uploaded to the mAdb database (Microarray Database, a collaboration of the Center for Information Technology/BioInformatics and Molecular Analysis Section (CIT/BIMAS) and National Cancer Institute/Center for Cancer Research (NCI/CCR) at the National Institutes of Health (NIH); see http://nciarray.nci.nih.gov/) and formatted via the export function for use with Biometric Research Branch (BRB) ArrayTools (developed by Dr. Richard Simon and the BRB ArrayTools Development Team, National Cancer Institute, Frederick, MA, USA).

### 2.4 Analysis of gene expression

Data from 4 independent experiments and 3 untreated controls were used for all statistical analyses. Expression analyses were performed using BRB ArrayTools. Data were background‐corrected, flagged values were removed, spots in which both signals were <100 were filtered out, ratios were log base 2‐transformed, and Lowess intensity‐dependent normalization was applied to adjust for differences in labelling intensities of the Cy3 and Cy5 dyes.^22^ Analysis was restricted to genes present on >50% of the arrays after filtering. The gene expression profile of all treatment groups was compared with that of the untreated control groups. A *P* value cut‐off of 0.0001 was used to identify genes whose expression was significantly up‐regulated after CpG ODN stimulation when compared with controls. Data were evaluated using Ingenuity Pathway Analysis (IPA; Ingenuity Systems Inc., Redwood City, CA, USA). Ingenuity Pathway Analysis maps each gene within a global molecular network derived from information contained in the Ingenuity Pathways Knowledge Base. A “network” in IPA is defined as a graphical representation of the molecular relationships among specific genes, represented as nodes, and the biological relationship between nodes shown as a connecting line. All connections are supported by published data stored in the Ingenuity Pathways Knowledge Base and/or PubMed database. Ingenuity Pathway Analysis ranks all genes based on their connectivity, using a generalization of the concept of node degree, which measures the number of single genes to which a gene is connected (see https://analysis.ingenuity.com/pa/info/help/Ingenuity_Network_Algorithm_Whitepaper_FINAL(2).pdf and for details Ref. 23).

### 2.5 Statistical analysis

Genes that were expressed differentially in the treatment groups were identified using a random‐variance *t* test. The random‐variance *t* test is an improvement over the standard, separate *t* test, as it permits sharing information among genes about within‐class variation without assuming that all genes have the same variance.^24^ The behaviour of specific gene subsets and functional groups was analysed with the IPA Comparison Tool. Ingenuity Pathway Analysis does provide a denominator for each functional group. Based on the entity of genes known for a specific function and the entity of genes significantly regulated in a data set, the probability that the regulation of a specific functional group is different from a random distribution is calculated. Differences between regulators in terms of number of genes regulated were established by chi‐square analysis and Fisher's exact test. Differences in fold changes between groups of genes were established by *t* test analysis (2‐sided).

To identify biological functions expected to increase or decrease according to the observed gene expression changes in the data set, we used the IPA Downstream Effects analytic. For each biological function, a statistical quantity was computed, called the activation *z*‐score. The activation *z*‐score is used to infer likely activation states of biological functions based on comparison with a model that assigns random regulation directions (for more information see http://pages.ingenuity.com/rs/ingenuity/images/0812%20downstream_effects_analysis_whitepaper.pdf).

## 3 RESULTS

### 3.1 Temporal pattern and functional groups of gene regulation 24 hours following myocardial I/R in CpG ODN 1668‐treated mice

We previously demonstrated that I/R following pre‐treatment with CpG ODN 1668 induced early pronounced myocardial gene activation (30 minutes to 3 hours following I/R) with selected induction of specific functional groups, compared to pre‐conditioning with control ODN.^12^ In this present work, we focused on genetic profile changes at a later time‐point, 24 hours after I/R. Both the PBS and the control ODN groups showed only a moderate increase in differential gene expression (165 and 126 genes, respectively), whereas in the sham group (surgical procedure without subsequent pre‐conditioning or I/R), 89 genes were differentially regulated, allowing us to determine the effects on gene expression elicited by induction of I/R alone. Differential gene expression in the CpG ODN group, showed a reduced number of regulated genes compared to the 3 hours time‐point, yet they still remained at a high activation level (434 genes, *P* < .0001 compared to control ODN group) and reflected the effect of CpG ODN 1668. Gene groups mediating “cellular movement” or “cellular function and maintenance” were among the top scoring functional categories, depicted in detail in Table 1. These groups primarily focus on the restoration of cellular integrity.

**Table 1:** Top scoring molecular, cellular and physiological development functions at 24 h post‐I/R in CpG‐treated animals

Molecular, cellular and physiological development functions	*P* value
Cellular movement	2.79E‐31
Immune cell trafficking	2.78E‐31
Haematological system development and function	3.31E‐27
Tissue morphology	8.04E‐24
Cellular function and maintenance	9.47E‐22
Cell‐to‐cell signalling and interaction	3.67E‐19
Cell death and survival	4.41E‐16
Tissue development	3.38E‐15
Cardiovascular system development and function	5.07E‐15

Table shows up‐regulated functions (according to IPA definition) 24 h post‐I/R in CpG‐treated animals, graded by significance of up‐regulation (Fisher's exact test; the *P* value indicates the probability that the biological process category is enriched in this microarray experiment by chance).

CpG ODN 24 h, *P* < .0001 data set, up‐regulated genes.

In order to validate our murine cardiac I/R model, we evaluated toxicity functions as a quality control. Toxicity functions in IPA relate to organ dysfunction and mark symptomatic pathological end‐points. The gene expression pattern identified “cardiac infarction” (*P* < 2.26E‐11), “cardiac dysfunction” (*P* < 1.18E‐6) and “cardiac inflammation” (*P* < 1.96E‐5) to be highly significantly induced (supplementary data, Table S1).

### 3.2 CpG ODN 1668‐dependent, prolonged modulation of signal transduction pathways after myocardial I/R

**Figure 2:**
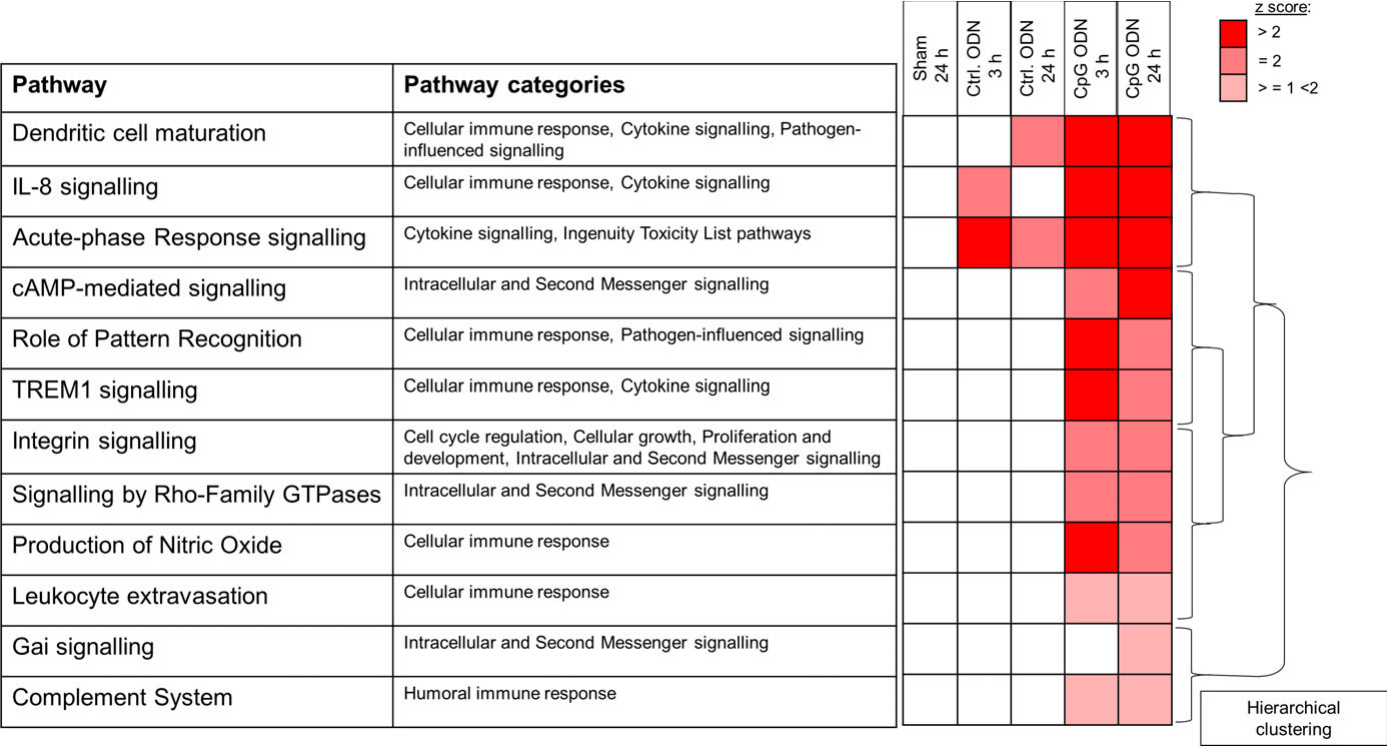
Prolonged up‐regulation of defined pathways 3 h towards 24 h post‐I/R is a CpG‐mediated effect. Figure shows heat map for up‐regulated pathways in control and CpG ODN‐treated animals, 3 and 24 h post‐I/R. Hierarchical clustering according to IPA definition. Heat map colour‐coded according to the *z*‐score (see 2 section)

Next, we analysed the CpG ODN 1668‐mediated gene expression profile of defined signal transduction pathways 24 hours after murine myocardial I/R. We sought to identify specific genetic changes we presumed might mediate the protective effects of TLR9 pre‐conditioning. I/R without preceding TLR9 activation (ie following pre‐conditioning with control ODN) resulted in very modest changes in pathway activation, which began to decline after 24 hours (Figure 2). In contrast, CpG pre‐treatment prompted strong activation of several defined pathways. This stimulation even increased activation along these pathways (ie “cAMP‐mediated signalling”) or in other cases at least sustained activation at high levels over the prolonged observation period of 24 hours after I/R (eg “dendritic cell maturation”, “IL‐8 signalling” and “acute‐phase response signalling”). Some pathways (“Gai signalling”) instead showed a delayed activation pattern following pre‐conditioning with CpG, not yet detectable 3 hours after I/R. Lastly, CpG‐mediated activation status of some of the pathways began to decline after 24 hours (eg “role of pattern recognition”, “TREM1 signalling” and “production of nitric oxide”). Of note, none of the pathways were altered in the sham group.

In contrast to these pathway activations, several pathways, described as mediating important proinflammatory responses, were instead suppressed at 24 hours, despite having initially been up‐regulated at 3 hours following I/R in the CpG ODN 1668 group. These include interferon (IFN) signalling, IL‐6 signalling, TLR signalling and NF‐κB signalling (Table 2).

**Table 2:** Down‐regulation of proinflammatory pathways 3 h towards 24 h post‐I/R following CpG pre‐conditioning

Pathway	−log (*P* value) 3 h	−log (*P* value) 24 h	Delta −log (*P* value)
Interferon signalling	11.39	2.54	8.85
IL‐6 signalling	8.52	1.19	7.33
Activation of IRF	6.37	0.41	5.96
Oncostatin‐M signalling	6.15	0.63	5.52
Toll‐like receptor signalling	5.71	0.35	5.36
PI3K signalling	4.56	0.25	4.31
NF‐κB signalling	4.13	0.40	3.73
P38‐MAPK signalling	4.08	0.63	3.45
HMGB1 signalling	3.27	1.81	1.46

Table shows up‐regulated pathways 3 and 24 h post‐I/R, respectively, in CpG‐treated animals. Significance is based on Fisher's exact test (the p value indicates the probability that the biological process category is enriched in this microarray experiment by chance).

IRF, interferon regulatory factor.

CpG ODN, *P* < .0001 data set, up‐regulated genes.

### 3.3 Genes predicted to increase survival of cardiomyocytes are strongly up‐regulated by CpG ODN 1668 pre‐conditioning at 24 hours post‐I/R

The extent of irreversible ischaemic damage in the early phase after acute MI is clearly and independently associated with adverse ventricular remodelling and worse patient outcome.^25^ According to IPA analysis, several genes known to influence cell survival were strongly induced at 24 hours post‐I/R in the CpG pre‐treatment group, compared to control. The *P* value in IPA for this functional group was 8E‐09, implying that the difference in regulation between these groups group is far away from a random distribution. Of these significantly up‐regulated genes, 75% have been described as increasing cell survival, for example, BCLA1, MT2, CTGF, Saa3 and Usp17la. Another subgroup of these highly up‐regulated genes includes those known to decrease the rates of death of cardiomyocytes, for example, TIMP1, HSPB1, NAD+ and APOD (Table 3).

**Table 3:** Genes predicted to increase cardiomyocyte survival are strongly up‐regulated by CpG pre‐conditioning 24 h post‐I/R

Genes in data set	Expression fold change
TIMP1^a^	37.000
SAA3	15.082
USP17la	9.598
MT2	9.400
CCL2	7.798
SELL	7.387
BCL2A1	6.426
APOD^a^	6.303
CTGF	5.773
POSTN	5.529
COL1A1	5.423
HSPA1A/HSPA1B	4.791
PLAC8	4.684
HSPB1^a^	4.500
FCER1G	4.441
NAD+^a^	4.362
TYROBP	4.306
ITGAM	3.570
FCGR1A	3.480
ITGB2	3.442
S1PR2	3.319
CHI3L1	2.880
MFAP5	2.855
CEACAM1	2.779
CD44	2.765
MT1	2.667
IRF9	2.574

Table shows significantly up‐regulated genes 24 h post‐I/R in CpG‐treated animals, graded by fold change of expression.

a Genes known to decrease cell death of cardiomyocytes.

CpG ODN 24 h, *P* < .0001 data set, up‐regulated genes.

### 3.4 Genes mediating cardiovascular angiogenesis are strongly up‐regulated by CpG ODN 1668 pre‐conditioning at 24 hours post‐I/R

**Figure 3:**
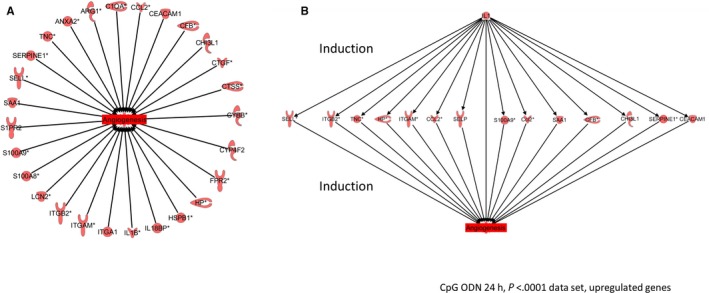
Genes predicted to increase cardiovascular angiogenesis are strongly up‐regulated by CpG pre‐conditioning 24 h post‐I/R. (A) Network analysis of CpG ODN‐induced activation of angiogenic genes. Ingenuity Pathway Analysis was used to identify the network of regulatory genes (open labels) contributing to CpG ODN‐induced activation. (B) IL1 complex is one regulator of angiogenesis 24 h post‐I/R. Figure shows network of genes significantly induced by IL1 complex. These genes are known to increase cardiovascular angiogenesis (see also Figure 3A). Asterisks indicate that fold change values of replicates have been averaged

Angiogenesis is a key factor in long‐term restoration of a sufficient blood supply to the myocardium. Twenty‐four hours post‐I/R, genes known to regulate cardiovascular angiogenesis were markedly up‐regulated in the CpG ODN 1668 pre‐conditioning group compared to control. Of these up‐regulated genes, 82.4% have been described to increase cardiovascular angiogenesis. A complete overview is given in Figure 3A (see also Table S2).

Specific upstream regulators induce these aforementioned genes. On this study, we identified the IL1 complex as one regulator of the network acting to increase cardiovascular angiogenesis at 24 hours in the CpG pre‐conditioning group (Figure 3B), including all of the most significantly activated genes from Figure 3A.

### 3.5 CpG ODN 1668‐mediated, IL‐10‐dependent modulation of gene expression

**Figure 4:**
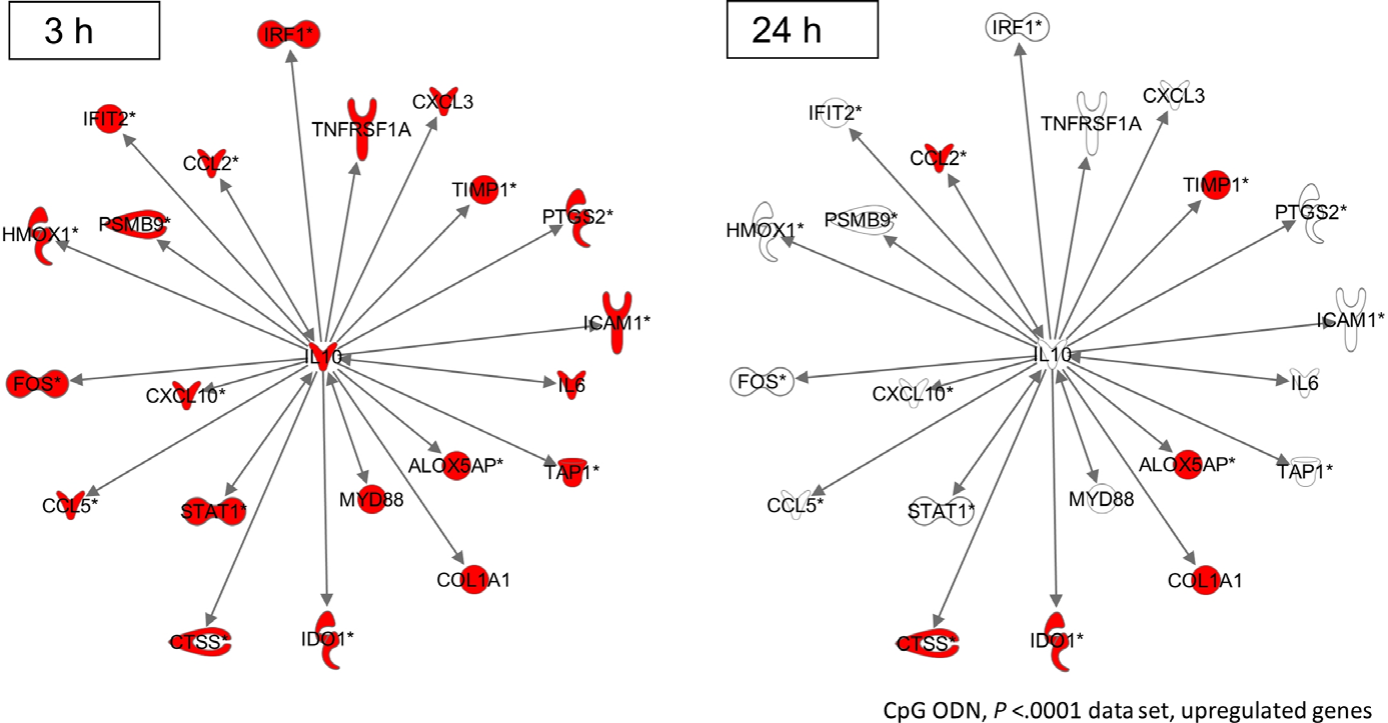
CpG‐mediated IL‐10 activity effectively down‐regulates target genes (3 h vs 24 h). Figure shows target genes of IL‐10 network in CpG‐treated animals. While 3 h post‐I/R, all genes were strongly up‐regulated, most genes experience down‐regulation towards 24 h post‐I/R. Asterisks indicate that fold change values of replicates have been averaged

We previously demonstrated that induction of IL‐10 plays an important role in the cardioprotection of CpG ODN 1668.^12^ We, therefore, specifically analysed the network of genes, which are thought to be modulated by IL‐10 as an upstream regulator. At 3 hours following I/R, these primarily proinflammatory genes were significantly induced along with IL‐10 (Figure 4). Follow‐up of the IL‐10 network at 24 hours post‐I/R indicated, indeed, 80% of the selected genes were actually suppressed by CpG pre‐conditioning.

## 4 DISCUSSION

Bacterial DNA contains CG motifs that interact with TLR9. The ensuing innate immune response is characterized by the production of a variety of Th1 and proinflammatory cytokines, as well as the maturation and proliferation of immune cells.^26^ Synthetic oligodeoxynucleotides (ODN) expressing CpG motifs mimic that immunostimulatory activity of bacterial DNA.^11^, ^26^ The signalling pathway triggered upon interaction of CpG with TLR9 necessitates, among other things, the recruitment of MyD88 and the stimulation of several mitogen‐activated kinases and transcription factors.^27^ Previous studies have identified diverse changes in gene expression following CpG stimulation.^28^, ^29^ In vivo studies demonstrated that CpG treatment triggers a rapid increase in gene activation with mRNA levels largely returning to baseline after 2‐3 days.^29^

Pre‐conditioning using immunological active ligands for the Toll‐like receptor 9 may ameliorate cell and tissue damage induced by myocardial I/R. However, the underlying genetic principles remain unclear. This study sought to identify the time course of certain functional pathways and cardiac gene expression profiles in a murine model of acute myocardial infarction after pre‐conditioning with CpG. We found that TLR9 pre‐conditioning results in profound induction of differential gene expression: strengthening protective functions and pathways, while limiting the impact of potentially detrimental genetic activity.

We have previously demonstrated that pre‐treatment with the CpG ODN 1668 16 hours before induction of I/R in mice could effectively reduce the size of the resulting infarct as well as rates of functional cardiac failure.^12^ Heightened up‐regulation of IL‐10 is supposed to be one key factor. Additional microarray analyses of gene expression revealed potent early induction of proinflammatory functional groups and pathways. However, as essential end‐points of this therapeutic approach to minimizing myocardial injury, such as infarct size or the preservation of cardiac function, can only be shown long‐term (24‐72 hours up to several days), gene expression analyses should be performed at later stages as well, assuming that these end‐points actually have a correlate at the level of gene expression.

### 4.1 TLR9‐mediated modulation of processes initiating and maintaining cardiac I/R injury

CpG pre‐conditioning induces early, yet sustained differential regulation of myocardial gene expression. Several pathways, such as IL‐8 signalling or acute‐phase reaction, reflect a TLR9‐induced inflammatory response, which are still active more than 24 hours after pre‐treatment and I/R, respectively. However, injury following I/R is a complex process involving ion accumulation, generation of free radicals, endothelial dysfunction, as well as immune activation, not all of which necessarily have concomitant changes in gene expression.^1^, ^30^ For example, the intracellular accumulation of Na^+^ ions is a result of the depletion of the cell's ATP pool, causing the energy‐dependent ion pumps to cease their activity. Nonetheless, the biocomputational analysis with IPA allowed us to identify several pathways being involved in the development of I/R injury. They are differentially regulated after pre‐conditioning with CpG ODN 1668, with heightened up‐regulation early after myocardial I/R and with a late‐phase decline after 24 hours.^12^ IFN‐γ, acting as an inflammatory cytokine, secreted by endothelial cells and leucocytes, is thought to contribute to the no‐reflow phenomena following reestablishment of coronary perfusion, which lead to sustained myocardial damage.^31^, ^32^ In keeping with this, our analyses revealed, after an initial up‐regulation, the subsequent suppression of the IFN‐γ pathway 24 hours following I/R, mediated by pre‐treatment with CpG. The latter also effected a decline in activation of associated interferon regulatory factors (IRFs), which are directly involved in the development of heart dysfunction and cell death following myocardial I/R injury.^33^ Numerous studies have demonstrated involvement of signalling via the transcription factor NF‐κB in progression of I/R‐induced myocardial injury.^34^ NF‐κB activation precipitates the synthesis and secretion of IL‐6. Murine closed‐chest IL‐6 knockout models of myocardial infarction, similar to the one used in our study, exhibit attenuated I/R damage.^35^ Both NF‐κB and IL‐6 signalling were, after an initial increase, among the pathways shown to be suppressed 24 hours following I/R in animals pretreated with CpG. On the other hand, Oncostatin‐M (OSM), a cytokine also belonging to the IL‐6 group and, therefore, promoted by NF‐κB activation, shows a similar pattern of activity in CpG‐pretreated mice. OSM, too, seems to rather confer protective effects in myocardial I/R. Indeed, early up‐regulation of OSM expression contributes to macrophage recruitment via induction of MCP‐1, thus initiating reparative processes.^36^

### 4.2 TLR9‐mediated modulation of tissue‐protective processes during cardiac I/R

As depicted above, pre‐conditioning using TLR9 stimulation exerts an amelioratory influence on myocardial I/R injury via suppression of proinflammatory pathways at 24 hours. However, our findings suggest that the induction of genes mediating tissue repair and restoration of myocardial integrity might be paramount in conferring the benevolent effects of CpG. This comprises genes associated with either tissue development or cellular function and maintenance. While I/R following pre‐conditioning with the control ODN displayed a very weak influence on promoting potentially protective pathways, CpG pre‐treatment resulted in early and sustained activation of safeguarding signal transduction mechanisms. Signalling activity involving cAMP and protein kinase A (PKA) was increased in CpG‐pretreated mice and exerted protective effects in myocardial I/R.^37^ Dendritic cells, whose maturation was boosted by TLR9 pre‐conditioning, infiltrated the infarcted heart and bolstered healing and restorative processes by controlling recruitment of monocytes and macrophages.^38^

CpG pre‐treatment exclusively induced the up‐regulation of a number of genes 24 hours after I/R that are implicated in lower rates of myocardial cell death. Specifically, we identified TIMP1, APOD and NAD+, among others, to have been highly propagated.^39^, ^40^, ^41^ It is known that TIMP1 inhibits metalloproteinases and it was shown to directly mediate protection from I/R injury in a rat model of myocardial infarction.^39^ Other genes, such as BCLA1, MT2, Saa3 and Usp17la, have likewise been demonstrated to exercise positive influences on cell survival.^42^, ^43^, ^44^, ^45^, ^46^

The activation of angiogenesis represents another requisite aspect of restoration initiated after myocardial ischaemic injury. Increasing angiogenesis in infracted tissue was demonstrated to occur after pre‐conditioning using CpG ODNs, restoring myocardial function after I/R in a very recent study by Zhou et al.^47^ In line with this, our study identified a set of genes with specific implications for the fostering of angiogenesis to be significantly up‐regulated in TLR9‐pretreated mice. Among the leading up‐regulated genes we discovered were S100A9, S100A8, ARG1, CFB and CCL2, all of which have been demonstrated to exert specific positive influences on cardiovascular angiogenesis.^48^, ^49^, ^50^, ^51^ Moreover, using IPA, we identified the IL1 gene complex as one regulator of increased cardiovascular angiogenic activity 24 hours following I/R in the CpG‐pretreated animals, which influenced the target genes mentioned above. The IL1 complex contains genes coding for both anti‐ and proinflammatory cytokines, including IL‐1A, IL‐1B and IL‐1RA.^52^ Indeed, gain‐of‐function experiments could demonstrate that overexpression of IL‐1RA mediates protection after myocardial I/R.^53^

As our previous analyses revealed, positive clinical effects of CpG pre‐conditioning are accompanied by an heightened up‐regulation of IL‐10 activity, suggesting that IL‐10 is a key regulator of TLR9‐driven cardioprotection.^12^ Several other publications have confirmed this basic finding, demonstrating it not only after the use of CpG, but also following ischaemic, vagal or pharmacologic (eg using morphine) pre‐ or post‐conditioning.^9^, ^54^, ^55^ With our recent work, we tracked changes in the IL‐10 network 24 hours after myocardial I/R to identify their specific influence on downstream gene regulation. After being initially induced along with IL‐10, a number of primarily proinflammatory genes were down‐regulated at this later time‐point owing to the suppressive influence of IL‐10. Numerous IL‐10‐dependent genes have been described to afford cardioprotective effects when suppressed. Inhibition of STAT1, a nuclear transcriptional factor involved in the induction of cardiomyocyte apoptosis, provides effective protection from myocardial I/R injury.^56^ The contribution of diverse chemokines to I/R injury, involving CCL2, CCL5, CXCL3 and CXCL10, and the protective effect of their selective suppression, respectively, have been demonstrated in a number of publications.^57^, ^58^, ^59^, ^60^ This also applies for the transcriptional controllers FOS and HMOX1.^57^

Several other studies have shown a protective effect of CpG pre‐conditioning for several other inflammatory conditions—some authors identifying similar underlying effects of CpG pre‐conditioning to those found in our study. For example, Vartanian et al^61^ demonstrated that CpG alters the regulation of cerebral microRNAs (miRNAs), inducing genomic reprogramming that mediates protection against cerebral ischaemic injury. A benevolent effect of CpG on acute lung inflammation was found by Moon et al.^62^ In that study, CpG stimulated matrix protein CCN1 secretion via the BiP/GRP78‐Src(Y527)‐JNK‐Cav‐1(Y14) pathway. This, in turn, promoted anti‐inflammatory IL‐10 release from epithelial cells, subsequently suppressing TNF‐α and macrophage inflammatory protein (MIP) 2 secretion as well as neutrophil infiltration in the lungs. In a murine model of acute polymicrobial sepsis, Weighardt et al^63^ showed that that administration of CpG prior to induction of sepsis resulted in an enhanced effector cell response of innate immunity. Hence, CpG pre‐conditioning may, therefore, help prevent sepsis‐associated immunoparalysis. Finally, Mathur et al^64^ demonstrated that pre‐conditioning with CpG greatly attenuated LPS‐mediated cardiac dysfunction in a murine model. Gene expression analysis revealed an up‐regulation of the NF‐κB pathway inhibitors TNFAIP3, NFKBIA, TRIM30 and TNIP1.

Obviously, our study has limitations. The IL1 complex prompting of increased cardiovascular angiogenic activity 24 hours following I/R in CpG‐pretreated animals was not verified using blocking or knockdown/knockout approaches in this study, and, hence, remains speculative. Moreover, although we had previously found a cardioprotective impact of CpG‐mediated IL‐10 up‐regulation by blocking IL‐10R1, we did not repeat these experiments in this study. However, taken together, our data reveal an early, yet prolonged induction of differential gene expression by pre‐conditioning with CpG ODN. The bolstering of numerous protective pathways and functions and the, presumably mainly IL‐10‐mediated, suppression of proinflammatory activity may be the genetic correlate of the clinically observed cardioprotective effects of CpG pre‐conditioning.

## CONFLICT OF INTEREST

The authors confirm that there are no conflict of interests.
