# Supplementary material for: Synthetic CpG oligonucleotides induce a genetic profile ameliorating murine myocardial I/R injury
Source: J Cell Mol Med. 2018 Apr 19;22(7):3397–407. doi: 10.1111/jcmm.13616 (PMC6010716; doi:10.1111/jcmm.13616)
Supplement: Supplementary file 1 [file JCMM-22-3397-s001.docx]

***Supplementary data***

***Table S1*: Top scoring toxicity functions at 24 hrs post I/R**

| **Toxicity functions** | **p value** |
| --- | --- |
| Cardiac infarction | 2.26E-11 |
| Cardiac dysfunction | 1.18E-06 |
| Cardiac inflammation | 1.96E-05 |

Table shows upregulated functions with cardiotoxic implications (according to IPA definition) 24 hrs post I/R, graded by significance of upregulation (Fisher’s exact test; the p value indicates the probability that the biological process category is enriched in this microarray experiment by chance).

CpG ODN 24 hrs, p <0.0001 dataset, upregulated genes

***Table S2*: Genes predicted to increase cardiovascular angiogenesis are strongly upregulated by CpG preconditioning 24 hrs post I/R**

| **Genes in dataset** | **expression fold change** |
| --- | --- |
| S100A9 | 40.364 |
| S100A8 | 30.735 |
| ARG1 | 21.312 |
| CFB | 8.929 |
| CCL2 | 8.786 |
| HP | 7.907 |
| SELL | 7.387 |
| IL1B | 6.094 |
| C1QA | 6.000 |
| TNC | 6.000 |
| FPR2 | 5.909 |
| CTGF | 5.773 |
| SERPINE1 | 5.536 |
| SAA1 | 4.750 |
| LCN2 | 4.705 |
| HSPB1 | 4.500 |
| IL18BP | 4.181 |
| CTSS | 4.164 |
| ANXA2 | 3.706 |
| ITGAM | 3.570 |
| ITGB2 | 3.442 |
| S1PR2 | 3.319 |
| CYBB | 2.909 |
| CHI3L1 | 2.880 |
| CEACAM1 | 2.779 |
| ITGA1 | 2.733 |
| CYP4F2 | 2.462 |

Table shows upregulated genes 24 hrs post I/R in CpG-treated animals, graded by fold change of expression.

CpG ODN 24 hrs, p <0.0001 dataset, upregulated genes
